# Difficulties Faced by Vietnamese Migrants in Japan in Accessing Healthcare During the COVID-19 Pandemic and Their Self-Reported Health Perceptions

**DOI:** 10.7759/cureus.74058

**Published:** 2024-11-19

**Authors:** Tadashi Yamashita, Pham Nguyen Quy, Emi Nogami, Chika Yamada, Kenji Kato

**Affiliations:** 1 Department of Nursing, Kobe City College of Nursing, Kobe, JPN; 2 Department of Medical Oncology, Kyoto Min-iren Central Hospital, Kyoto, JPN; 3 Department of Social Welfare, School of Psychology and Social Welfare, Mukogawa Women’s University, Nishinomiya, JPN; 4 Department of Environmental Coexistence, Center for Southeast Asian Studies, Kyoto University, Kyoto, JPN; 5 Department of Nursing, Kobe Women’s University, Kobe, JPN

**Keywords:** covid-19, health care, health perceptions, japan, pandemics, vietnam

## Abstract

Background: The COVID-19 pandemic disproportionately affected migrant populations in Japan, including Vietnamese migrants, who faced distinct challenges in accessing healthcare compared to native Japanese citizens. These challenges, exacerbated by the structural complexities of Japan’s healthcare system during the pandemic, likely influenced their subjective health perceptions. Nevertheless, studies on Vietnamese migrants’ difficulties in healthcare access during the pandemic and their perceived health perceptions are lacking. To address this gap, the present study aimed to clarify the relationship between healthcare access difficulties and subjective health perceptions among this migrant group in Japan during the COVID-19 pandemic.

Methods: A repeated cross-sectional design was employed, targeting Vietnamese migrants aged 18 years and older living in Japan. From 2021 to 2023, annual online surveys were conducted to assess demographics, COVID-19 history, healthcare access difficulties, and subjective health perceptions. A binary logistic regression analysis was performed to examine the relationship between healthcare access difficulties when suspected of contracting COVID-19 infection and subjective health perceptions.

Results: The number of survey participants totaled 631 in 2021, 214 in 2022, and 176 in 2023. The findings showed that in 2021, 26.1% of the respondents faced difficulties accessing healthcare when suspected of contracting COVID-19 infection. This was significantly associated with poor subjective health perception (odds ratio {OR}: 2.35; 95% confidence interval {CI}: 1.15-4.79). In 2022, 13.6% of the respondents reported difficulties accessing healthcare, also significantly associated with poor subjective health perception (OR: 2.75; 95% CI: 1.02-7.45). In 2023, 10.2% of the respondents faced difficulties, but no significant association with subjective health perception was observed (OR: 2.18; 95% CI: 0.72-6.63).

Conclusions: Difficulties in accessing healthcare during the COVID-19 pandemic had a significant impact on the subjective health perceptions of Vietnamese migrants residing in Japan, particularly in the early stages of the pandemic. Therefore, to improve and ensure equitable access to healthcare services for migrant populations during public health crises, there is a need to implement targeted interventions that reduce the barriers for these vulnerable groups.

## Introduction

In general, the COVID-19 pandemic had a disproportionate effect on the health of migrants compared to that of the natives of a country. The pandemic exacerbated existing health disparities globally, with migrants facing heightened risks due to barriers to accessing and utilizing healthcare [1]. In this study, we focus on Vietnamese migrants in Japan, a group that, despite experiencing rapid population growth, remains a particularly vulnerable group [2]. Vietnamese migrants, predominantly younger in age, often lack sufficient socioeconomic foundations and have not yet attained adequate proficiency in the Japanese language [3]. This linguistic barrier further complicates their integration into Japanese society and limits their access to essential services. The healthcare challenges faced by Vietnamese migrants, specifically in navigating the Japanese healthcare system, differ from those encountered by native Japanese citizens and other migrant groups [4]. During the pandemic, the establishment of fever outpatient clinics and other specialized healthcare services added layers of complexity to the Japanese healthcare system, making it challenging even for Japanese citizens [5]. These complexities were particularly problematic for migrants with limited Japanese language proficiency, potentially exacerbating health disparities and increasing risks to their overall well-being.

Healthcare access has long been recognized as crucial for maintaining the health of migrant populations [6]. Compared to the pre-pandemic levels, access to healthcare for migrants worsened during the COVID-19 pandemic [7]. This decline in healthcare access had a significant impact on the subjective health perception of migrants, defined as their personal evaluation of their own health status. Restricted access to medical services and resources further compromised their health and well-being [8].

In Japan, the methods for seeking medical care during COVID-19 became increasingly complex, making it difficult for individuals infected with COVID-19 to access healthcare services, even when experiencing mild symptoms. This increased complexity in healthcare access likely influenced the subjective health perceptions of the population in Japan [9]. Previous studies have explored the subjective health and well-being of migrants in various contexts. Sieverding et al. examined the impact of the COVID-19 pandemic on subjective well-being in the Middle East and North Africa, revealing significant gender differences and underscoring the pandemic’s broader health impacts on subjective well-being [10]. Two years after the pandemic, Alarcão et al. found that the mental health and well-being of migrant populations in Portugal were significantly affected, emphasizing the importance of healthcare access and quality during crises [11]. Abbas et al. further highlighted the barriers to healthcare accessibility faced by migrants during the pandemic, elucidating the complex interplay between healthcare access and subjective health [12].

Despite the importance of healthcare access for migrant populations globally, especially since the pandemic, no studies have specifically examined the challenges faced by Vietnamese migrants in Japan in accessing healthcare during this period. Specifically, documentation is lacking on whether these migrants encountered challenges in accessing medical care if they contracted COVID-19. Furthermore, existing research does not address whether these healthcare access difficulties during the pandemic were associated with their subjective health perceptions. Therefore, this study aimed to address his gap in the literature by investigating whether healthcare access difficulties during the COVID-19 pandemic significantly influenced the subjective health perceptions of Vietnamese migrants in Japan.

## Materials and methods

Study design

To investigate the difficulties Vietnamese migrants in Japan faced in accessing medical care for a possible COVID-19 infection and its impact on their subjective health, this study employed a repeated cross-sectional design. This approach was chosen to capture changes over time within the same population during the COVID-19 pandemic.

Subjects and methods

The study targeted Vietnamese migrants, aged 18 years or older, residing in Japan. Convenience sampling was employed, leveraging an online questionnaire developed using SurveyMonkey (Niskayuna, NY: Momentive Inc.) and distributed via Facebook (Menlo Park, CA: Meta Platforms Inc.) to groups frequented by Vietnamese migrants in Japan. This sampling method, chosen for its practicality and wide reach, is particularly suited to the constraints of the COVID-19 pandemic. However, this approach carried the possibility of selection bias, as it may have excluded individuals without internet access or those less comfortable with digital technology. Individuals unable to independently respond to the questionnaire and those with short-term residency status were excluded from the study.

**Figure 1:**
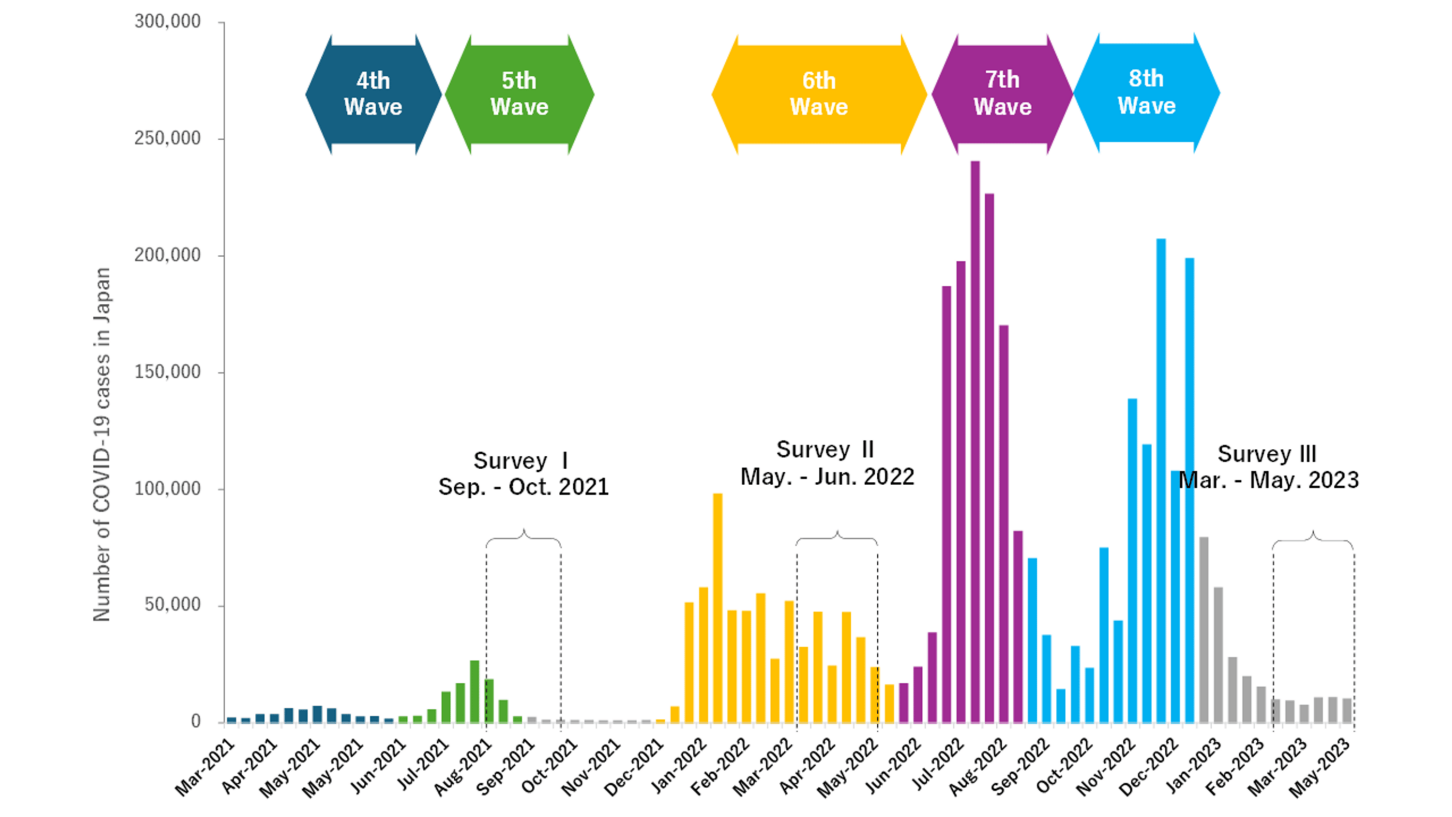
Trends in COVID-19 case numbers from the fourth to eighth waves in Japan, including the current survey period.

The cross-sectional surveys were conducted annually from 2021 to 2023 using an online, self-administered questionnaire. The first survey (Survey I) was conducted from September to October 2021, the second (Survey II) from May to June 2022, and the third (Survey III) from March to May 2023, during the COVID-19 pandemic in Japan (Figure 1). Survey I received 1,084 responses, with 631 valid responses after excluding incomplete submissions (effective response rate: 58.2%); Survey II received 284 responses, of which 214 were valid responses (effective response rate: 75.4%); Survey III received 356 responses, of which 176 were valid responses (effective response rate: 49.4%).

Measures

The self-administered questionnaire encompassed items related to participant demographics (gender, age, years of residence in Japan, and residency status), COVID-19-related questions (history of COVID-19 infection and difficulties in accessing medical care for a possible COVID-19 infection), and health status (subjective health perception). Residency status was categorized as follows: permanent resident, spouse of a Japanese national, spouse of a permanent resident, Indochinese refugee, family stay, engineer/specialist in humanities/international services, professor, researcher, educator, designated activities (including household workers under economic partnership agreements), international student, technical intern trainee, specified skilled worker, and no response. For analytical purposes, residency status was reclassified into the following three groups to differentiate between students and workers: (1) international student, (2) non-student (including permanent residents, workers, etc.), and (3) no response.

Responses to the question regarding difficulties in accessing medical care for a possible COVID-19 infection (difficulties in accessing medical care when suspected of COVID-19 infection: DAC-COVID) were provided using a five-point Likert scale with a score of 0 indicating “not at all” difficult to 5 indicating “highly difficult.” Prior to this study, a pretest of the DAC-COVID questionnaire item was conducted to ensure that the researchers' intent was clearly understood. For this study’s analysis, responses regarding DAC-COVID were classified into two categories as follows: “slightly,” “moderately,” “very” and “highly” difficult to access healthcare were combined into reported, while “not at all” difficult to access healthcare was classified as not reported. This item related to DAC-COVID was included due to the challenges resulting from the establishment of fever clinics and the complexity of medical consultation methods in Japan, which hindered patients’ access to appropriate care [13].

The reliability and validity of the psychometric properties of the four-point Likert-type scale used for assessing subjective health perception in this study were then evaluated [14]. For this study, subjective health perceptions were classified into two categories as follows: “poor” and “very poor” were combined as poor, while “good” and “very good” were combined into good. The questionnaire was administered entirely in Vietnamese, with translation experts conducting back-translation to enhance its reliability.

Statistical analysis

Descriptive statistics were employed to calculate the proportions, means, and standard deviations of each variable for each year. An unadjusted bivariate logistic regression model and a binary logistic regression model were created, with subjective health perception as the dependent variable, to elucidate the relationship between subjective health perception and difficulties in accessing medical care for a possible COVID-19 infection. The dependent variable (subjective health perception) was binary, with “poor” coded as 0 (reference) and “good” coded as 1 for analytical purposes.

The selection of independent variables was based on the objectives of this study, as well as relevant findings from other studies, ensuring no multicollinearity existed among the explanatory variables [15]. First, a bivariate logistic regression analysis was conducted to examine the relationship between the dependent variable and each explanatory variable. Subsequently, a binary logistic regression model analysis was performed using the forced entry method. To enhance model reliability, the number of explanatory variables in the binary logistic regression model was minimized [16]. The final explanatory variables included “age,” “length of stay in Japan,” “difficulties in accessing healthcare when suspecting a COVID-19 infection,” and “history of COVID-19 infection.” “Gender” was excluded from the final model, as it did not significantly influence the model in multiple iterations. It should be noted that demographic variables, such as gender, do not always need to be included in every analysis [17]. Additionally, owing to the small number of responses in the “no response” category for residency status, these were treated as missing values in the logistic regression analysis, following the recommendations of Cornilly et al. [17]. A significant level of 5% (two-sided) was applied for all analyses, which were conducted using SPSS version 26.0 (Armonk, NY: IBM Corp.).

Ethical considerations

The participants provided their consent by marking a checkbox at the beginning of the online survey. The survey ensured the anonymity of the participants by not collecting personally identifiable information, including email addresses. The analysis included only those who marked the consent checkbox and completed the survey. This study was approved by the Kobe City College of Nursing Research Ethics Review Committee (#20124-05).

## Results

In the 2021 survey, a total of 9.0% (n = 57) of the participants had a history of COVID-19 infection. DAC-COVID was reported by 26.1% (n = 165), whereas 73.8% (n = 466) did not have any difficulties. Finally, 5.9% (n = 37) rated their health as poor, while 94.1% (n = 594) rated their health as good. In the 2022 survey, a history of COVID-19 infection was reported by 18.2% (n = 39), while 81.8% (n = 175) had no such history. DAC-COVID was reported by 13.6% (n = 29), and 86.5% (n = 185) not reporting any difficulties. Poor health was reported by 12.6% (n = 27) of the participants, while 87.4% (n = 187) reported good health. In the 2023 survey, a history of COVID-19 infection was reported by 46.6% (n = 82), while 53.4% (n = 94) had no such history. DAC-COVID was reported by 10.2% (n = 18), and 89.8% (n = 158) did not face any difficulties. Poor health was reported by 22.7% (n = 40), while 77.3% (n = 136) rated their health as good (Table 1).

**Table 1:** Characteristics of the study participants in 2021, 2022, and 2023. DAC-COVID: difficulties in accessing medical care when suspected of COVID-19 infection

Characteristics		2021 (n = 631)		2022 (n = 214)		2023 (n = 176)	
		n	%	n	%	n	%
Age (years)	Mean±SD	26.01±4.69		26.83±4.41		32.16±10.39	
Gender	Male	352	55.8	112	52.3	71	40.3
	Female	275	43.6	102	47.7	105	59.7
	Other	4	0.6	0	0	0	0
Residence status	Student	185	29.3	65	30.4	35	19.9
	Non-student workers/residents	421	66.7	143	66.8	138	78.4
	No response	25	4.0	6	2.8	3	1.7
Years in Japan (year)	Mean±SD	3.41±3.13		4.35±2.53		7.49±10.27	
A history of COVID-19 Infection	Infected	57	9.0	39	18.2	82	46.6
	Not infected	574	91.0	175	81.8	94	53.4
DAC-COVID	Reported	165	26.1	29	13.6	18	10.2
	Not reported	466	73.8	185	86.5	158	89.8
Subjective health status	Poor	37	5.9	27	12.6	40	22.7
	Good	594	94.1	187	87.4	136	77.3

The results of the unadjusted bivariate logistic regression model on the relationship between subjective health perception and various variables are summarized as follows. In 2021, a statistically significant association was observed between subjective health perception and DAC-COVID (unstandardized partial regression coefficient {UPRC}: 0.702; odds ratio {OR}: 2.02; 95% confidence interval {CI}: 1.02-3.99). In 2022, residency status was significantly associated with subjective health perception (UPRC: 0.834; OR: 2.30; 95% CI: 1.01-5.23), whereas DAC-COVID was not associated (UPRC: 0.965; OR: 2.62; 95% CI: 1.00-6.92). In 2023, the length of stay in Japan was significantly associated with subjective health perception (UPRC: -0.032; OR: 0.97; 95% CI: 0.94-0.99), whereas DAC-COVID remained non-significant (UPRC: 0.601; OR: 1.82; 95% CI: 0.64-5.22) (Table 2).

**Table 2:** Results of an unadjusted bivariate logistic regression model with subjective health perception of Vietnamese migrants in Japan as the dependent variable. DAC-COVID: difficulties in accessing medical care when suspected of COVID-19 infection; OR: odds ratio; CI: confidence interval

Items		2021			2022			2023		
		Odds ratio (95% CI)	Unstandardized coefficients	p-Value	Odds ratio (95% CI)	Unstandardized coefficients	p-Value	Odds ratio (95% CI)	Unstandardized coefficients	p-Value
Age (year)		1.01 (0.94- 1.09)	0.012	0.74	1.07 (0.97-1.18)	0.066	0.20	0.96 (0.93-0.99)	-0.044	0.01
Years in Japan (year)		0.95 (0.88-1.03)	-0.050	0.24	1.24 (0.98-1.56)	0.214	0.07	0.97 (0.94-0.99)	-0.032	0.04
Residency status	Student	1.00 (reference)	-0.141	0.71	1.00 (reference)	0.834	0.046	1.00 (reference)	-0.712	0.17
	Non-student workers/residents	0.87 (0.41-1.84)			2.30 (1.01-5.23)			0.49 (0.18-1.36)		
DAC-COVID	Reported	1.00 (reference)	0.702	0.04	1.00 (reference)	0.965	0.051	1.00 (reference)	0.601	0.26
	Not reported	2.02 (1.02-3.99)			2.62 (1.00-6.92)			1.82 (0.64-5.22)		
History of COVID-19 infection	Infected	1.00 (reference)	0.213	0.70	1.00 (reference)	0.288	0.57	1.00 (reference)	0.047	0.90
	Not infected	1.24 (0.42-3.63)			1.33 (0.50-3.56)			1.05 (0.52-2.12)		

The results of an adjusted binary logistic regression model between subjective health perception and various variables indicated that participants who did not experience difficulties in accessing healthcare during the COVID-19 pandemic had higher odds of reporting better subjective health perceptions compared to those who faced such difficulties (UPRC: 0.854; OR: 2.35; 95% CI: 1.15-4.79). A history of COVID-19 infection was not significantly associated with subjective health perception (UPRC: 0.486; OR: 1.63; 95% CI: 0.53-4.95). Similar trends were observed for 2022. Participants who did not face difficulties in accessing healthcare for a possible COVID-19 infection had higher odds of reporting better subjective health (UPRC: 1.013; OR: 2.75; 95% CI: 1.02-7.45). No significant association was found between a history of COVID-19 infection and subjective health perception (UPRC: 0.214; OR: 1.24; 95% CI: 0.45-3.41). For 2023, the analysis did not reveal any significant relationship between not facing difficulties in accessing healthcare for a possible COVID-19 infection and subjective health perception (UPRC: 0.780; OR: 2.18; 95% CI: 0.72-6.63). Similarly, the history of COVID-19 infection remained non-significant in relation to subjective health perception (UPRC: 0.122; OR: 1.13; 95% CI: 0.54-2.35) (Table 3).

**Table 3:** Results of an adjusted binary logistic regression model with subjective health perception of Vietnamese migrants in Japan as the dependent variable. Adjusted for baseline score of “age,” “length of stay in Japan,” “DAC-COVID,” and “history of COVID-19 infection.” DAC-COVID: difficulties in accessing medical care when suspected of COVID-19 infection; OR: odds ratio; CI: confidence interval

Items		2021			2022			2023		
		Odds ratio (95% CI)	Unstandardized coefficients	p-Value	Odds ratio (95% CI)	Unstandardized coefficients	p-Value	Odds ratio (95% CI)	Unstandardized coefficients	p-Value
Age (year)		1.05 (0.95-1.15)	0.047	0.34	0.97 (0.86-1.10)	-0.032	0.61	0.96 (0.92-1.01)	-0.036	0.09
Years in Japan (year)		0.90 (0.81-1.00)	-0.104	0.06	1.21 (0.93-1.57)	0.190	0.15	0.99 (0.95-1.03)	-0.008	0.68
Residency status	Student	1.00 (reference)	-0.274	0.52	1.00 (reference)	0.716	0.15	1.00 (reference)	-0.408	0.47
	Non-student workers/residents	0.76 (0.33-1.77)			2.05 (0.78-5.38)			0.66 (0.22-2.02)		
DAC-COVID	Reported	1.00 (reference)	0.854	0.02	1.00 (reference)	1.013	0.046	1.00 (reference)	0.780	0.17
	Not reported	2.35 (1.15-4.79)			2.75 (1.02-7.45)			2.18 (0.72-6.63)		
History of COVID-19 infection	Infected	1.00 (reference)	0.486	0.39	1.00 (reference)	0.214	0.68	1.00 (reference)	0.122	0.74
	Not infected	1.63 (0.53-4.95)			1.24 (0.45-3.41)			1.13 (0.54-2.35)		

## Discussion

This study examined the relationship between difficulties in accessing healthcare for a possible COVID-19 infection and the subjective health perception of Vietnamese migrants in Japan over three consecutive years (2021-2023). The results indicated that a significant proportion of the participants faced challenges in obtaining medical care for a possible COVID-19 infection. A statistically significant association was found between difficulties in accessing healthcare and poor subjective health perceptions for the years 2021 and 2022; however, this association was not significant in 2023. This implies that during the initial stages of the COVID-19 pandemic, migrants in Japan faced difficulties accessing healthcare, which may have impacted their health. To prepare for a future pandemic, it is crucial to establish systems and strategies to ensure that migrants in Japan consistently have access to appropriate medical care.

Our findings align with those of previous studies, which indicate that access to healthcare significantly impacts subjective health perception [18]. Studies have shown that perceived barriers to healthcare can lead to delayed care, reduced use of preventive services, and poor health outcomes [19]. The significant association observed in 2021 and 2022 suggests that the initial stages of the pandemic may have exacerbated existing barriers to healthcare for Vietnamese migrants, consistent with the findings on other migrant populations [20]. The lack of significant association in 2023 may indicate a progressive adaptation or improvement in healthcare access for this group over time.

Several mechanisms could explain the observed association between difficulties in healthcare access and subjective health perception. Firstly, the uncertainty and stress associated with not knowing how to seek medical care for a possible COVID-19 infection could directly affect mental health and overall well-being [21]. Secondly, limited access to healthcare may lead to untreated conditions, exacerbating physical health problems and negatively impacting subjective health perception [22].

The number of COVID-19 cases in Japan surged dramatically from 2021 to 2023, straining the healthcare system and leading to temporary restrictions on medical consultations [23]. However, as the pandemic continued, these restrictions were gradually eased, allowing better accessibility to healthcare services. This improvement in healthcare accessibility could explain the reduced association between healthcare access difficulties and subjective health perception in 2023. The initial high barriers in 2021 and 2022 likely contributed to greater stress and worse health perceptions, and the subsequent relaxation of restrictions may have alleviated some of these issues [24-26]. Additionally, language barriers, lack of familiarity with the healthcare system, and discrimination could further hinder migrants’ access to care and affect their health perceptions [27]. Specific interventions, such as offering multilingual information and support services, providing cultural competency training for healthcare providers, and implementing anti-discrimination policies, should be implemented to address these barriers.

This study highlights the critical role of healthcare accessibility in shaping health perceptions among Vietnamese migrants in Japan. It underscores the need for targeted interventions to improve healthcare accessibility for migrant populations, particularly during public health crises. The repeated cross-sectional design of this study, spanning three years, provides valuable insights into the evolving challenges faced by this population and the potential long-term impacts on their health perception.

Despite its strengths, this study has several limitations. First, reliance on self-reported data may introduce response bias, particularly in sensitive areas such as health status and healthcare access. Second, the use of online surveys may have introduced selection bias by excluding individuals without Internet access or those who are less comfortable with digital technology. Third, the cross-sectional nature of the annual surveys limits the ability to establish causality between healthcare access difficulties and subjective health perception. Future research should consider employing longitudinal methods to track individual changes over time. Fourth, the study design, which involved repeated cross-sectional surveys conducted once per year, may introduce bias. Given that the sample groups varied annually, bias might have arisen, particularly in 2023, when the respondents had a longer average duration of residence in Japan compared to those from the 2021 and 2022 samples. This difference in residency duration may have influenced the results, thereby impacting the comparability of the findings across the years. Fifth, the potential underrepresentation of specific subgroups within the Vietnamese migrant community may limit the generalizability of the results. Finally, the variation in sample sizes across different survey years may have influenced the consistency and reliability of the outcomes. Future studies should aim to address these limitations by incorporating more robust sampling techniques and potentially increasing the frequency of data collection to capture individual-level changes more effectively.

## Conclusions

This study contributes to the existing literature by providing evidence that difficulties in accessing healthcare for a possible COVID-19 infection significantly impacted the subjective health perceptions of Vietnamese migrants in Japan, especially during the early stages of the pandemic. The findings underscore the critical need for equitable access to healthcare services to maintain the overall health and well-being of migrant populations during public health emergencies. Specific interventions, such as targeted communication strategies and healthcare support tailored to linguistic and cultural needs, are essential for reducing barriers and ensuring equitable healthcare access. Future research should address the study’s limitations, including the use of online surveys and the cross-sectional nature of the data, to further explore the long-term impact of healthcare access difficulties on migrants’ health perceptions. Policymakers and healthcare providers must prioritize the development and implementation of strategies aimed at improving healthcare accessibility for these vulnerable groups, particularly in preparation for potential pandemics and public health crises.
